# **A 33-year-old Man with Abdominal Pain**

**Published:** 2016

**Authors:** Kuo-Chih Chen, Aming Chor-Ming Lin, Chin-Chu Wu, Tzong-Luen Wang, Chai-Hock Chua

**Affiliations:** 1 Emergency Department, Shin Kong Wu Ho-Su Memorial Hospital, Taipei, Taiwan.; 2 Department of Intensive Care Unit, Shin Kong Wu Ho-Su Memorial Hospital, Taipei, Taiwan.; 3 School of Medicine, Fu-Jen Catholic University, New Taipei City, Taiwan.; 4 Department of Medical Imaging, Shin Kong Wu Ho-Su Memorial Hospital, Taipei, Taiwan.; 5 Division of Cardiovascular Surgery, Department of Surgery, Shin Kong Wu Ho-Su Memorial Hospital, Taipei, Taiwan.

## Case presentation:

**Figure 1:**
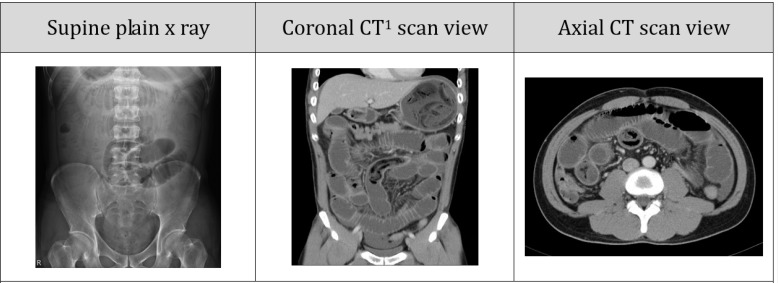
Patient’s abdominal imaging.

A 33-year-old man presented to the emergency department ED) with complaint of 2-day history of abdominal pain. His pain developed with gradual onset prominently in epigastric area after eating dried mushrooms. The pain was diffuse, persistent, radiating to the back and aggravated by meal. He had been tolerating only liquids and had complaints of nausea and vomiting. He had no history of diabetes mellitus, hypertension, alcohol consumption, malignancy, or prior surgery. On arrival his blood pressure was 128/72 mmHg, with a heart rate of 101 beats/minute and a respiratory rate of 20 breaths/minute. He was afebrile. Physical examination revealed diffuse abdominal distention, hyper-pitched bowel sounds, and tenderness more marked over the umbilicus with no guarding or rebound tenderness. A complete blood cell count showed the following: leukocyte count 12600 /mm^3^; segmented neutrophils 90%; hemoglobin level of 14 mg/dl; hematocrit 30%; and platelet 420000/µL. Other laboratory studies included: glucose 101 mg/dL; serum urea nitrogen 45 mg/dL; serum creatinine 2.0 mg/dL; sodium 148 mEq/L; potassium 3.1 mEq/L; serum glutamic oxaloacetic transaminase (SGOT) 38 U/L and lipase 30 U/L. Figure 1 shows patient’s plain upright abdominal X-ray as well as coronal and axial cuts of abdominal CT scan. 

**What is your diagnosis?**

**Diagnosis:**

**Figure 2:**
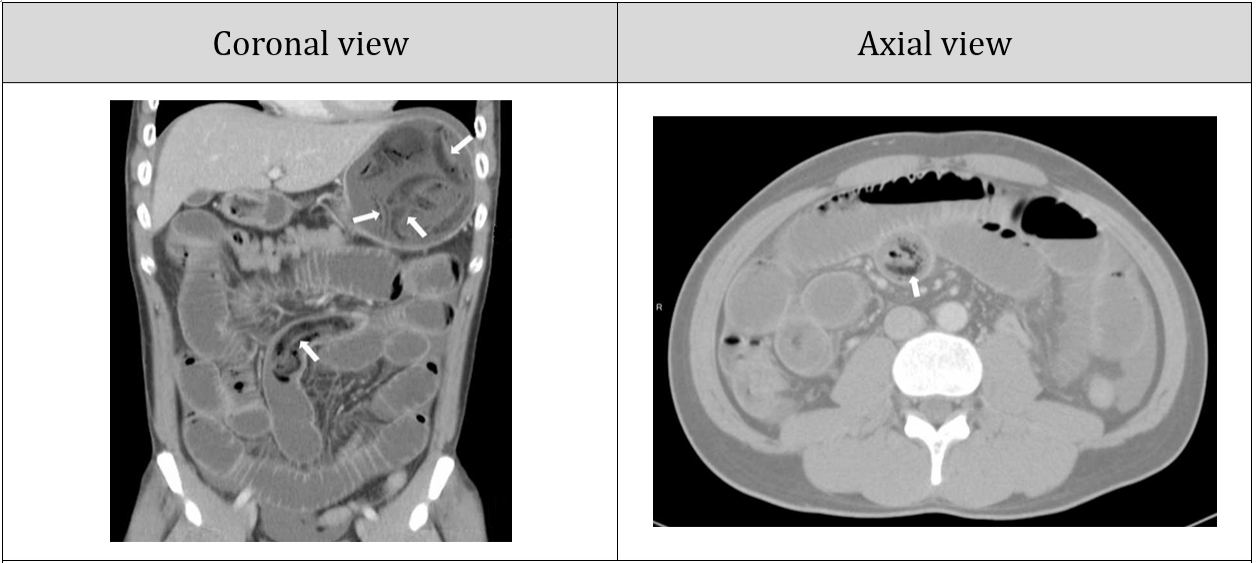
Patient’s abdominal computed tomography scan

Abdominal CT scan showed multiple masses with peculiar shape (Figure 2, arrows) in stomach and ileum. Post contrast CT scan showed peculiar shape masses impacting the bowel on the middle side of the abdomen with dilated small intestine above that level. The CT scan finding was consistent with evidence of a mechanical obstruction.

1: Computed tomography.

**Case fate:**

The patient underwent conservative treatment such as nasogastric suction, decompression, aggressive intravenous fluids, bowel rest and antibiotics for 3 days. The undigested mushrooms had passed through feces later. He had an uneventful recovery with no complications.

## Discussion:

Intestinal obstruction is a relatively common problem encountered in the ED, accounting for an estimated 15% of all emergency admissions for abdominal pain (-). Delayed diagnosis of small bowel obstruction is still associated significant and morbidity and mortality. Early diagnosis and identification of the cause of obstruction has importance in therapeutic management (5). 

The diagnosis may be suspected based upon clinical history, presentation, physical examination and radiologic findings. Abdominal pain and distention is the hallmark of all forms of intestinal obstruction, and constipation, nausea and vomiting are the most common symptoms. Tympany to percussion and hyper-pitched bowel sounds are the classic physical examination findings (6, 7). 

Although adhesion band and incarcerated hernia are among the most common causes of small bowel obstruction, bezoars and ingested materials could be considered as less common causes (1, 8). Bezoars are concretions of indigested or partially digested material in the gastrointestinal tract which divided to different types including phytobezoars, trichobezoars, pharmacobezoars and lactobezoars. An important cause of phytobezoars is dried fruits (9, 10). Predisposing factors include previous gastric surgery, inadequate chewing, excessive consumption of fruits rich in fibers, renal insufficiency, hypothyroidism, and chronic constipation (11). 

The initial evaluation of patients with clinical signs and symptoms of intestinal obstruction should included plain upright abdominal radiography. Abdominal CT scan can help to confirm the diagnosis of small bowel obstruction and identify strangulation and perforation complicating small bowel obstruction (12, 13). The bezoar could be seen on the CT scan examination. Intestinal obstruction caused by bezoar not only requires immediate treatment but also recognition of underlying cause of bezoar formation (14, 15). The presence of peritoneal irritation signs usually indicates late obstruction with complications, including vascular compromise or perforation. Failure to resolve with adequate bowel decompression is an indication for surgical intervention. The findings of peritonitis, clinical instability, persistent abdominal pain are concerning for intra-abdominal sepsis, intestinal ischemia, or perforation, which mandate immediate surgical exploration (16). 

## References

[1] Jackson PG, Raiji M (2011). Evaluation and management of intestinal obstruction. American family physician.

[2] Kariman H, Shojaee M, Sabzghabaei A, Khatamian R, Derakhshanfar H, Hatamabadi H (2014). Evaluation of the Alvarado score in acute abdominal pain. Ulus Travma Acil Cerrahi Derg.

[3] Majidi A, Mahmoodi S, Baratloo A, Mirbaha S (2014). Atypical Presentation of Massive Pulmonary Embolism, a Case Report. Emergency.

[4] Forouzanfar M, Hatamabadi H, Hashemi B, Majidi A, Baratloo A, Shahrami A (2014). Outcome of nonspecific abdominal pain in the discharged patients from the emergency department. Journal of Gorgan University of Medical Sciences.

[5] Schwab DP, Blackhurst DW, Sticca RP, Laws II HL (2001). Operative acute small bowel obstruction: Admitting service impacts outcome/Discussion. The American surgeon.

[6] Mullan CP, Siewert B, Eisenberg RL (2012). Small bowel obstruction. American Journal of Roentgenology.

[7] Shin C-I (2015). Small Bowel Obstruction. Radiology Illustrated: Gastrointestinal Tract.

[8] Markogiannakis H, Messaris E, Dardamanis D, Pararas N, Tzertzemelis D, Giannopoulos P (2007). Acute mechanical bowel obstruction: clinical presentation, etiology, management and outcome. World journal of Gastroenterology.

[9] Gümüs M, Kapan M, Önder A, Tekbas G, Yagmur Y (2011). An unusual cause of small bowel obstruction: dried apricots. JPMA-Journal of the Pakistan Medical Association.

[10] Ooi S, Hong K (2015). Small bowel obstruction caused by dried apple. International journal of surgery case reports.

[11] Bedioui H, Daghfous A, Ayadi M, Noomen R, Chebbi F, Rebai W (2008). A report of 15 cases of small-bowel obstruction secondary to phytobezoars: predisposing factors and diagnostic difficulties. Gastroenterologie Clinique et biologique.

[12] Thompson JS (2002). Contrast radiography and intestinal obstruction. Annals of surgery.

[13] Xu G, Guo Y (2012). Computed Tomography Findings of Small Bowel Obstruction due to Bezoar Impaction: A Case Series. Emergency Medicine: Open Access.

[14] Wang P-Y, Wang X, Zhang L, Li H-F, Chen L, Wang X (2015). Bezoar-induced small bowel obstruction: Clinical characteristics and diagnostic value of multi-slice spiral computed tomography. World journal of gastroenterology: WJG.

[15] Porter DJ, Cosgrove C, Middleton E (2015). An Unusual Case of Small Bowel Obstruction. Journal of Medical Cases.

[16] Paulson EK, Thompson WM (2015). Review of small-bowel obstruction: the diagnosis and when to worry. Radiology.

